# Early Postoperative Inflammatory Responses Following Primary Total Hip Arthroplasty Using Different Antibiotic-Loaded Bone Cement Formulations: A Prospective Observational Cohort Study

**DOI:** 10.3390/jcm15176789

**Published:** 2026-09-01

**Authors:** Nicolas Catalin Ionut Ion, Sorin Radu Fleaca, Bogdan Axente Bocea, Cosmin Ioan Mohor, Mihai-Dan Roman, Călin-Ilie Mohor, Alexandru Florin Diconi, Alexandru Turcu, Iustin-Ilie Tutuianu, Mihai Faur, Vanesa-Maria Veres, Vicențiu-Vasile Vereș, Victoria Birlutiu

**Affiliations:** 1 Faculty of Medicine, Lucian Blaga University of Sibiu, Str. Lucian Blaga, Nr. 2A, 550169 Sibiu, Romania; nicolascatalinionut.ion@ulbsibiu.ro (N.C.I.I.); bogdanaxente.bocea@ulbsibiu.ro (B.A.B.); cosmin.mohor@ulbsibiu.ro (C.I.M.); mihairoman@ulbsibiu.ro (M.-D.R.); calin.mohor@ulbsibiu.ro (C.-I.M.); alexandru.diconi@ulbsibiu.ro (A.F.D.); alexandruss.turcu@ulbsibiu.ro (A.T.); iustin.tutuianu@ulbsibiu.ro (I.-I.T.); mihai.faur@ulbsibiu.ro (M.F.); vanesa.veres@ulbsibiu.ro (V.-M.V.); vicentiu.veres@ulbsibiu.ro (V.-V.V.); victoria.birlutiu@ulbsibiu.ro (V.B.); 2 County Clinical Emergency Hospital of Sibiu, 550245 Sibiu, Romania; 3 County Clinical Emergency Hospital of Cluj-Napoca, 010024 Cluj-Napoca, Romania

**Keywords:** hip, arthroplasty, antibiotic, intraoperative, gentamicin

## Abstract

**Introduction:** Periprosthetic joint infection (PJI) remains one of the most challenging complications following total hip arthroplasty (THA). Antibiotic-loaded bone cement (ALBC) provides high local antibiotic concentrations and is widely used in patients at increased risk of infection. Although several dual-antibiotic formulations have been investigated, their influence on the early postoperative inflammatory response has not been directly compared. **Methods:** In this prospective observational cohort study, 35 patients undergoing primary cemented THA between January 2025 and January 2026 were allocated to one of five ALBC regimens according to routine clinical practice: gentamicin alone, gentamicin–vancomycin, gentamicin–clindamycin, gentamicin–meropenem, or gentamicin–tobramycin (*n* = 7 per group). Serum C-reactive protein (CRP), erythrocyte sedimentation rate (ESR), and leukocyte count were measured preoperatively and on postoperative Days 1, 3, and 7. Longitudinal analyses accounting for within-patient correlations were performed to assess the effects of treatment group, postoperative time, and the group × time interaction. Day-specific between-group comparisons using one-way analysis of variance (ANOVA), with eta-squared (η^2^) as an effect-size measure, were performed as secondary analyses, with Bonferroni adjustment applied to post-hoc pairwise comparisons. **Results:** All treatment groups demonstrated progressive reductions in postoperative inflammatory markers. Longitudinal analysis demonstrated significant effects of treatment group, postoperative time, and the group × time interaction for CRP and ESR (all *p* < 0.001). For leukocyte count, significant effects of time and the group × time interaction were observed (both *p* < 0.001), whereas the overall group effect was not statistically significant (*p* = 0.071). Secondary day-specific analyses showed significant between-group differences in CRP, with comparatively lower values observed in the gentamicin–meropenem group. Several pairwise differences in CRP and ESR remained statistically significant after Bonferroni adjustment, whereas no pairwise differences in leukocyte counts remained significant after correction. **Conclusions**: Different antibiotic-loaded PMMA formulations were associated with distinct early postoperative inflammatory profiles. Gentamicin–meropenem was associated with comparatively lower CRP and ESR values; however, these exploratory findings do not establish superior infection-preventive efficacy and require confirmation in larger studies.

## 1. Introduction

Periprosthetic joint infection (PJI) remains one of the most devastating complications following total hip arthroplasty (THA), despite continuous advances in surgical techniques, implant design, perioperative management, and infection prevention strategies. Although the reported incidence after primary THA ranges from approximately 0.5% to 2%, PJI is associated with substantial patient morbidity, prolonged hospitalization, repeated surgical interventions, impaired functional outcomes, increased healthcare costs, and considerable socioeconomic burden [1,2]. Moreover, the increasing number of primary hip arthroplasties performed worldwide, together with an aging population characterized by multiple comorbidities, has resulted in a steady rise in the absolute number of PJIs. Patients with diabetes mellitus, chronic kidney disease, obesity, malignancy, autoimmune disorders, or long-term immunosuppressive therapy are particularly susceptible to postoperative infection, making effective perioperative prophylactic strategies essential for improving clinical outcomes and implant survival [1,3,4].

Antibiotic-loaded bone cement (ALBC) has become an established adjunctive strategy for preventing implant-related infections in cemented arthroplasty by delivering high local antibiotic concentrations directly at the bone–implant interface while minimizing systemic exposure and toxicity [5,6,7]. Polymethylmethacrylate (PMMA) acts as a carrier matrix that provides sustained antibiotic release, particularly during the early postoperative period when bacterial adhesion and biofilm formation are most likely to occur [6]. Gentamicin remains the most frequently incorporated antibiotic because of its broad antimicrobial spectrum, thermal stability during PMMA polymerization, and favorable elution characteristics [5]. To broaden antimicrobial coverage and potentially enhance antibiotic release through synergistic elution, gentamicin has increasingly been combined with additional agents such as vancomycin, clindamycin, tobramycin, and, more recently, meropenem [7,8,9,10]. Experimental and clinical studies have suggested that dual-antibiotic formulations may improve antimicrobial activity against resistant Gram-positive and Gram-negative microorganisms while maintaining acceptable mechanical properties of the cement when appropriate antibiotic concentrations are used [11,12,13,14].

Despite the widespread clinical use of antibiotic-loaded bone cement, several important questions remain unresolved. Most published investigations have focused on antibiotic elution kinetics, physicochemical properties of PMMA, microbiological efficacy, or the treatment of established periprosthetic joint infections using antibiotic spacers [15]. Comparatively fewer clinical studies have evaluated the biological response associated with different antibiotic-loaded cement formulations in patients undergoing primary THA. Early postoperative inflammatory biomarkers, including C-reactive protein (CRP), erythrocyte sedimentation rate (ESR), and leukocyte count, are routinely used to monitor postoperative recovery and may provide indirect information regarding the magnitude of the surgical inflammatory response. However, evidence directly comparing the postoperative evolution of these biomarkers among different dual-antibiotic bone cement formulations remains limited, particularly in patients presenting with multiple comorbidities that increase the risk of postoperative infectious complications. Consequently, no consensus currently exists regarding the optimal antibiotic combination for routine use in high-risk patients undergoing primary cemented THA. To our knowledge, this is among the first prospective observational studies to directly compare early postoperative inflammatory marker dynamics across five different antibiotic-loaded bone cement formulations in patients undergoing primary total hip arthroplasty.

The aim of this prospective observational cohort study was to compare the early postoperative inflammatory response among patients undergoing primary cemented total hip arthroplasty receiving five different antibiotic-loaded bone cement formulations: gentamicin alone, gentamicin–vancomycin, gentamicin–clindamycin, gentamicin–meropenem, and gentamicin–tobramycin. Postoperative changes in CRP, ESR, and leukocyte count were evaluated during the first postoperative week to determine whether different local antibiotic regimens were associated with distinct inflammatory profiles. We hypothesized that dual-antibiotic formulations would be associated with a more rapid postoperative decline in inflammatory markers than gentamicin-loaded cement alone.

## 2. Materials and Methods

This prospective observational cohort study was conducted at the Department of Orthopaedics and Traumatology, County Clinical Emergency Hospital, Sibiu, Romania, between January 2025 and January 2026. The study was designed to evaluate the early postoperative inflammatory response associated with different antibiotic-loaded bone cement (ALBC) formulations in patients undergoing primary cemented total hip arthroplasty (THA). The study protocol was approved by the Institutional Ethics Committee of the County Clinical Emergency Hospital, Sibiu (Approval No. 30673/11 November 2025), and all procedures were performed in accordance with the ethical principles of the Declaration of Helsinki and its subsequent amendments. Written informed consent was obtained from all participants before enrollment. The manuscript was prepared in accordance with the Strengthening the Reporting of Observational Studies in Epidemiology (STROBE) guidelines for observational studies.

Consecutive adult patients undergoing primary cemented THA during the study period were prospectively screened for eligibility. Patients were eligible for inclusion if they underwent their first THA procedure and presented with one or more clinically relevant comorbidities considered to increase perioperative risk. The comorbidity profile of the included cohort comprised type 2 diabetes mellitus, chronic kidney disease, arterial hypertension, and iron-deficiency anemia. Patients with active local or systemic infection before surgery, previous arthroplasty of the affected hip, known hypersensitivity to any antibiotic incorporated into the bone cement, refusal to participate, or incomplete postoperative laboratory follow-up were excluded from the study. A total of 35 patients fulfilled the eligibility criteria and were included in the final analysis.

Patients were allocated into five treatment groups according to the antibiotic-loaded bone cement used during routine clinical practice. Because antibiotic selection reflected the treating surgeon’s clinical judgment rather than random allocation, the present investigation should be regarded as an observational cohort study. Each treatment group included seven patients. Group A received gentamicin-loaded bone cement alone and served as the reference group. Group B received gentamicin combined with vancomycin, Group C received gentamicin combined with clindamycin, Group D received gentamicin combined with meropenem, and Group E received gentamicin combined with tobramycin.

The gentamicin–tobramycin regimen was included because this formulation was used in routine clinical practice during the study period rather than because the combination was expected to substantially broaden antimicrobial coverage. Both agents are aminoglycosides with the same principal mechanism of action and overlapping antibacterial spectra; therefore, this group should be regarded primarily as a dual-aminoglycoside formulation.

All surgical procedures consisted of primary cemented total hip arthroplasty performed by experienced orthopedic surgeons according to the institutional operative protocol. Commercial polymethylmethacrylate (PMMA) bone cement was used in all patients. Depending on the selected antibiotic regimen, either commercially available gentamicin-loaded Palacos^®^ R + G cement or Simplex P^®^ with tobramycin cement supplemented with powdered antibiotics was utilized. Because Palacos^®^ R + G and Simplex P^®^ with tobramycin have different manufacturer-specific recommendations for cement preparation and handling, each cement was prepared, mixed, and handled according to the instructions provided by its respective manufacturer rather than according to an identical mixing protocol. For the manually supplemented formulations, the additional antibiotic was incorporated in powdered form into the PMMA polymer powder before addition of the liquid monomer. The antibiotic was not additionally mortared, and fractionated admixing was not performed. The antibiotic and PMMA powders were manually mixed to obtain a macroscopically homogeneous distribution before addition of the liquid monomer. Subsequent mixing, handling, working time, and application followed the manufacturer-specific recommendations for the respective cement. The antibiotic-loaded cement was applied within the prepared femoral medullary canal and within the prepared acetabulum for fixation of the respective components according to standard cemented total hip arthroplasty technique.

Palacos^®^ R + G, containing 0.5 g gentamicin per 40 g PMMA, was used in Groups A–D, with 2 g vancomycin, 0.9 g clindamycin, or 1 g meropenem manually incorporated in Groups B–D, respectively. In Group E, Simplex P^®^ containing 1 g tobramycin per 40 g PMMA was used, with 0.5 g gentamicin manually incorporated. The cement formulations and exact antibiotic contents are summarized in Table 1 [15,16,17,18].

Peripheral venous blood samples were obtained preoperatively and on postoperative Days 1, 3, and 7 using standardized aseptic venipuncture techniques. Laboratory analyses were performed in the hospital’s accredited central laboratory according to institutional quality assurance protocols. The primary inflammatory biomarkers evaluated were serum C-reactive protein (CRP), erythrocyte sedimentation rate (ESR), and peripheral leukocyte count.

The primary outcome of the study was the temporal evolution of postoperative inflammatory markers during the first postoperative week following primary THA. Specifically, postoperative CRP, ESR, and leukocyte count were compared among the five antibiotic-loaded bone cement formulations to evaluate differences in the early inflammatory response. Because the study was designed to investigate postoperative inflammatory marker dynamics rather than clinical infection outcomes, microbiological cultures, implant survival, and long-term periprosthetic joint infection rates were not evaluated.

Given the exploratory nature of this prospective study, no formal sample size calculation was performed. Instead, all consecutive eligible patients treated during the predefined study period were included. Therefore, the findings should be considered hypothesis-generating and interpreted with caution until confirmed in larger prospective multicenter investigations. A sensitivity analysis based on the final sample size (*n* = 35, five groups) indicated 80% power at α = 0.05 to detect a large overall effect size of Cohen’s f ≈ 0.63.

**Figure 1:**
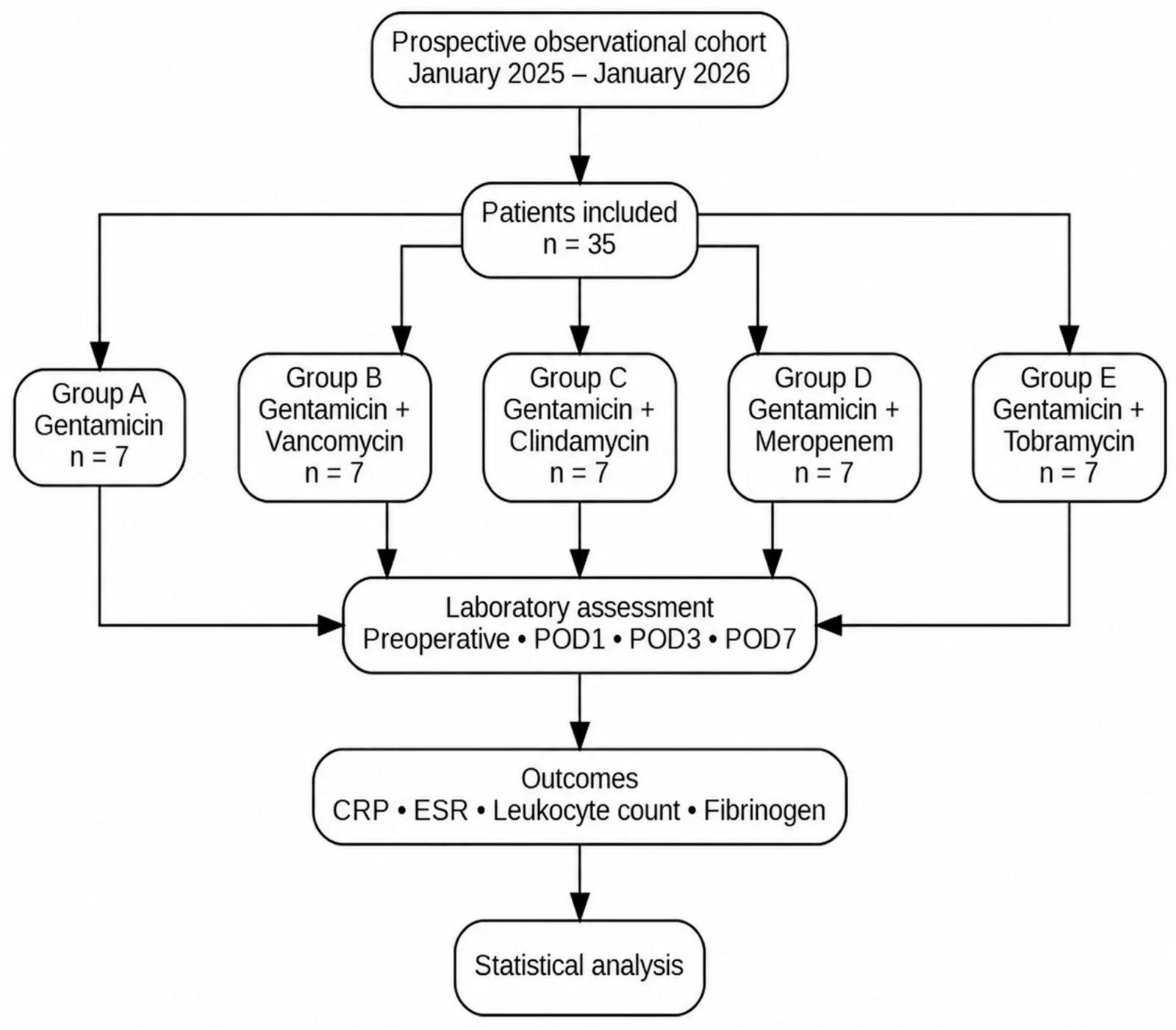
Flow diagram illustrating patient inclusion, allocation according to the antibiotic-loaded bone cement formulation used during routine clinical practice, postoperative laboratory assessments, and statistical analysis.

The study was designed and reported according to the Strengthening the Reporting of Observational Studies in Epidemiology (STROBE) statement. A flow diagram summarizing patient inclusion and study procedures is presented in Figure 1.

Statistical analysis was performed using IBM SPSS Statistics software (v28, IBM Corp., Armonk, NY, USA). Continuous variables are presented as mean ± standard deviation (SD), with 95% confidence intervals (CIs) where appropriate. Because CRP, ESR, and leukocyte counts were measured repeatedly in the same patients on postoperative Days 1, 3, and 7, longitudinal analyses were performed using a repeated-measures modeling approach to account for within-patient correlations. Treatment group, postoperative time, and the group × time interaction were included as fixed effects, with patient identification defining the repeated observations. Overall tests of fixed effects were performed to assess the effects of treatment group, time, and the group × time interaction for each inflammatory biomarker.

In addition to the primary longitudinal analysis, secondary between-group comparisons were performed separately at each postoperative time point (Days 1, 3, and 7) using one-way analysis of variance (ANOVA). For these secondary analyses, eta-squared (η^2^) was calculated as a measure of the overall between-group effect size, and 95% CIs were calculated for group means. The day-specific ANOVA results were interpreted as secondary analyses in the context of the overall longitudinal model and were not considered independent substitutes for the repeated-measures analysis. Statistical significance was defined as a two-sided *p*-value < 0.05. No multiplicity adjustment was applied to the omnibus ANOVA tests. When post-hoc pairwise comparisons between individual treatment groups were performed, Bonferroni adjustment was applied to account for multiple comparisons. The day-specific ANOVA and post-hoc results were considered secondary analyses and were interpreted in the context of the overall longitudinal model.

## 3. Results

A total of 35 patients undergoing primary cemented total hip arthroplasty were prospectively included in the study. All participants completed the planned postoperative laboratory evaluations on Days 1, 3, and 7, and no patients were lost during follow-up. Each treatment group consisted of seven patients according to the antibiotic-loaded bone cement formulation used during surgery. Baseline demographic and clinical characteristics of the five treatment groups are presented in Table 2. The groups showed broadly comparable distributions of age, sex, BMI, ASA status, and duration of intervention. No statistically significant between-group differences were identified for age (*p* = 0.535), BMI (*p* = 0.908), or duration of intervention (*p* = 0.229). All included patients were classified as ASA II. Relevant comorbidities, including type 2 diabetes mellitus, chronic kidney disease, hypertension, and iron-deficiency anemia, were distributed across the treatment groups. Given the small number of patients in each group, these baseline comparisons should be interpreted descriptively and do not exclude residual confounding.

Overall, all inflammatory biomarkers demonstrated the expected postoperative evolution, characterized by a gradual decline during the first postoperative week. However, differences in the magnitude of this decline were observed among the five antibiotic-loaded bone cement formulations.

### 3.1. Longitudinal Repeated-Measures Analysis

Longitudinal analysis accounting for repeated measurements within individual patients demonstrated significant effects of treatment group, postoperative time, and the group × time interaction on CRP and ESR (all *p* < 0.001). For leukocyte count, the effect of postoperative time and the group × time interaction were significant (both *p* < 0.001), whereas the overall treatment-group effect did not reach statistical significance (*p* = 0.071). The results of the repeated-measures analysis are summarized in Table 3.

### 3.2. C-Reactive Protein

**Figure 2:**
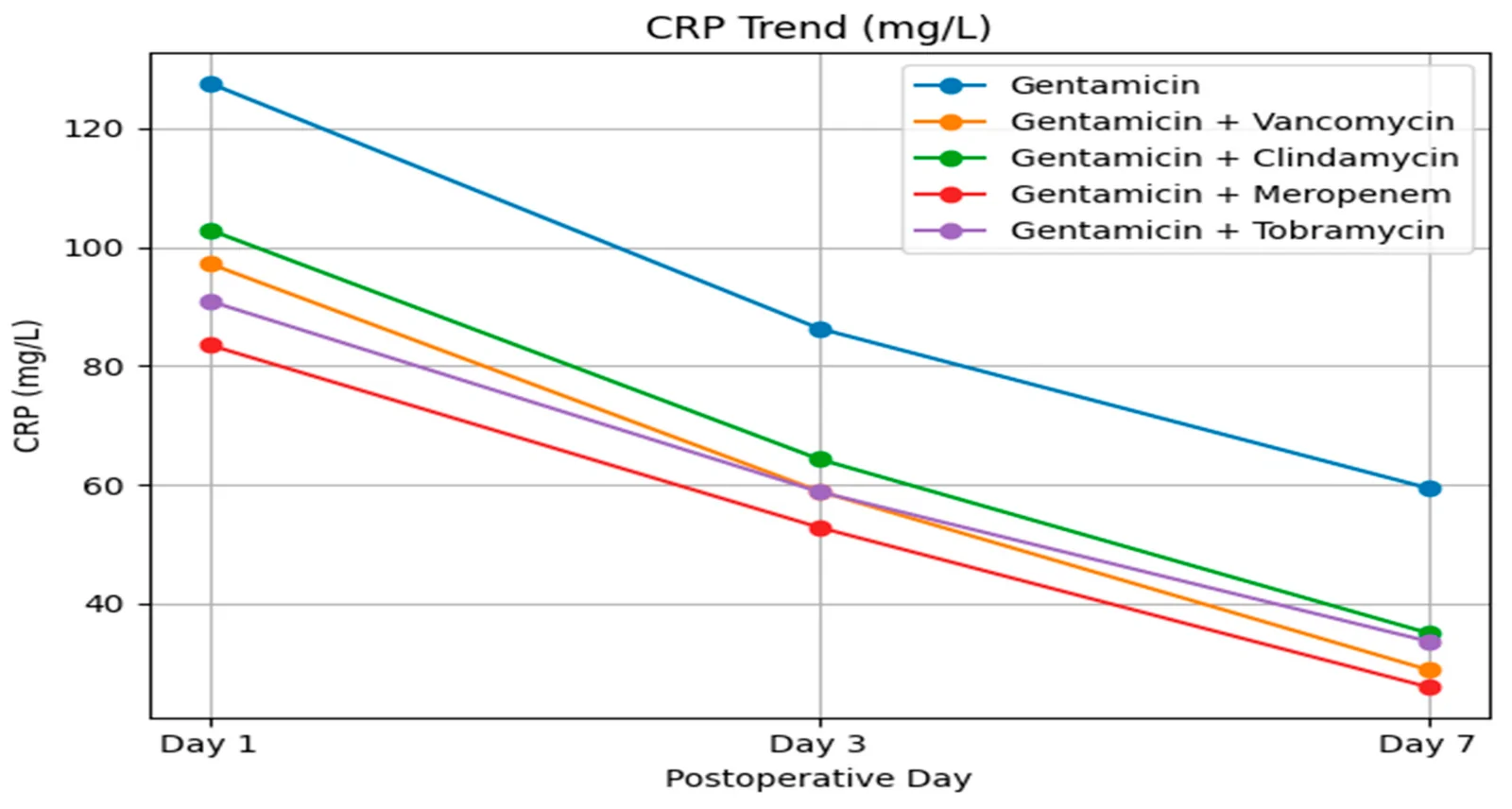
Postoperative CRP Dynamics in Patients Receiving Different Antibiotic-Loaded Bone Cements.

Mean postoperative CRP concentrations are presented in Table 4, while their temporal evolution is illustrated in Figure 2.

Significant differences in CRP levels were observed among treatment groups at all postoperative evaluations (Day 1, Day 3, and Day 7; all *p* < 0.001).

On postoperative Day 1, the highest mean CRP concentration was recorded in the gentamicin-only group (127.81 ± 11.70 mg/L), whereas the gentamicin–meropenem group demonstrated the lowest value (83.50 ± 2.32 mg/L). Intermediate CRP concentrations were observed in the gentamicin–tobramycin (90.91 ± 1.98 mg/L), gentamicin–vancomycin (97.17 ± 1.84 mg/L), and gentamicin–clindamycin (102.87 ± 2.36 mg/L) groups.

CRP concentrations decreased progressively in all treatment groups throughout the observation period. By postoperative Day 3, the gentamicin–meropenem group continued to demonstrate the lowest mean CRP value (52.76 ± 1.68 mg/L), followed by the gentamicin–tobramycin (58.76 ± 1.39 mg/L) and gentamicin–vancomycin (58.86 ± 2.42 mg/L) groups. The gentamicin-only group maintained the highest CRP concentration (86.39 ± 4.35 mg/L).

A similar pattern was observed on postoperative Day 7. The lowest mean CRP value remained in the gentamicin–meropenem group (25.93 ± 1.89 mg/L), followed by the gentamicin–vancomycin (28.77 ± 2.66 mg/L) and gentamicin–tobramycin (33.59 ± 1.15 mg/L) groups. The gentamicin-only group continued to present the highest CRP concentration (59.17 ± 13.02 mg/L).

### 3.3. Erythrocyte Sedimentation Rate

**Figure 3:**
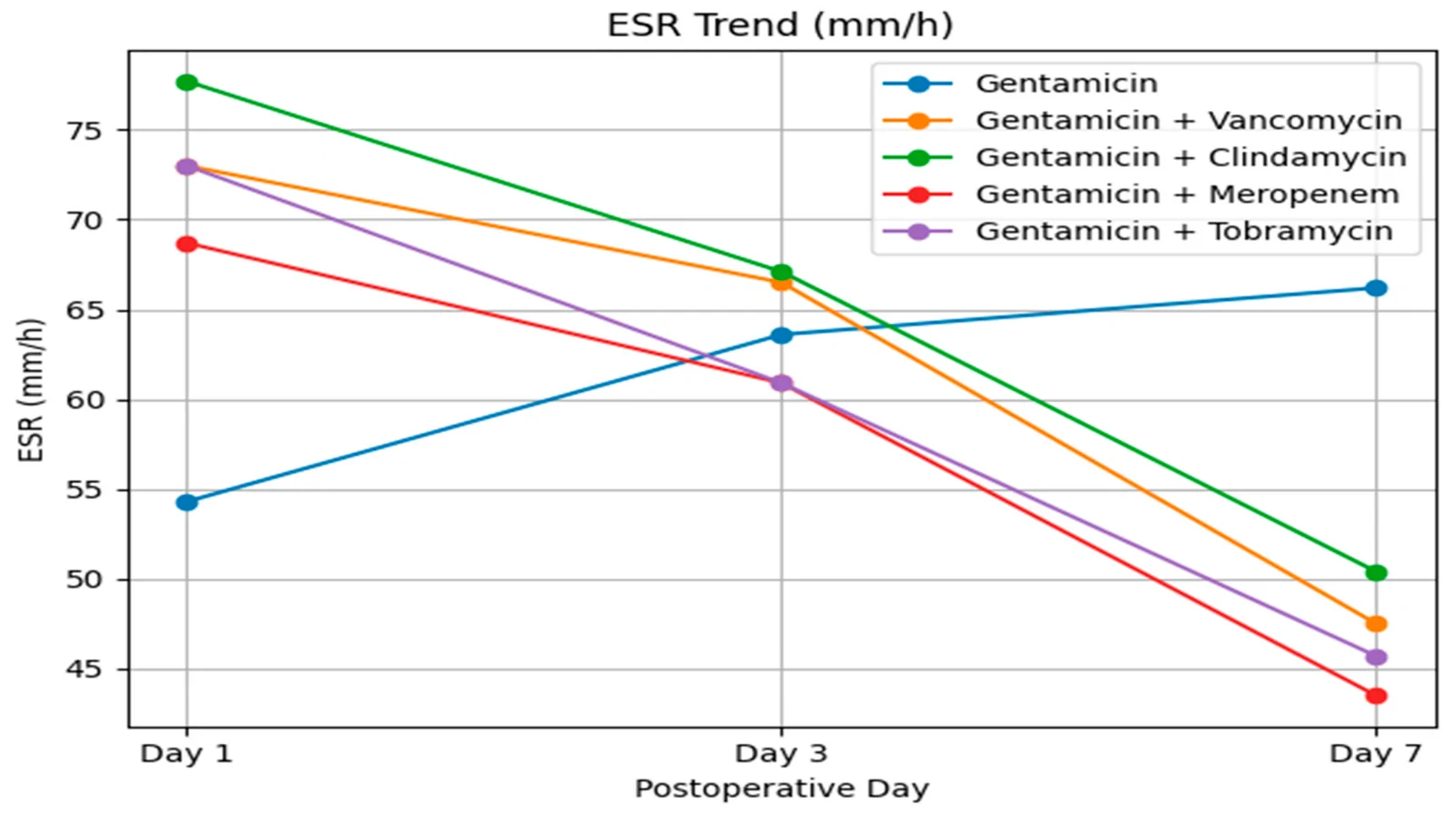
Postoperative ESR Dynamics by Antibiotic Cement Combination.

Mean ESR values are summarized in Table 5, and their postoperative evolution is presented in Figure 3.

Statistically significant differences in ESR were identified among treatment groups on postoperative Days 1 and 7 (both *p* < 0.001), whereas no significant between-group differences were observed on postoperative Day 3 (*p* = 0.099).

On postoperative Day 1, the gentamicin-only group exhibited the lowest mean ESR value (54.33 ± 4.42 mm/h), while the gentamicin–clindamycin group showed the highest value (77.74 ± 1.74 mm/h). The remaining combination regimens demonstrated intermediate values ranging from 68.67 ± 1.98 mm/h in the gentamicin–meropenem group to 73.97 ± 2.15 mm/h in the gentamicin–vancomycin group.

During postoperative follow-up, ESR values generally declined among patients receiving dual-antibiotic formulations. By postoperative Day 7, the gentamicin–meropenem group demonstrated the lowest mean ESR value (43.50 ± 3.03 mm/h), followed by the gentamicin–tobramycin (45.66 ± 2.55 mm/h) and gentamicin–vancomycin (47.43 ± 2.66 mm/h) groups. The gentamicin-only group presented the highest ESR value at the final evaluation (66.26 ± 8.56 mm/h).

### 3.4. Leukocyte Count

**Figure 4:**
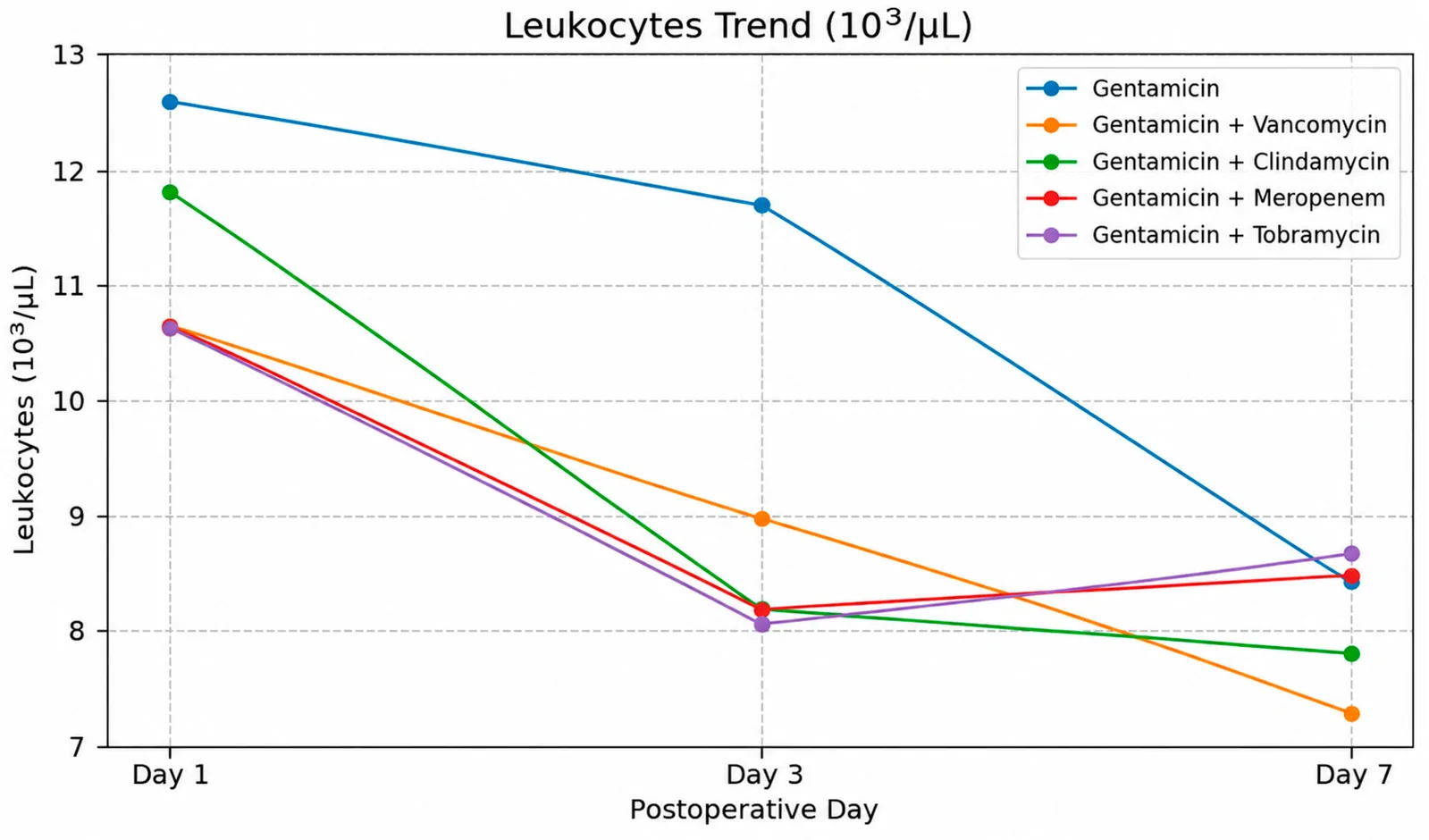
Postoperative Leukocyte Dynamics by Antibiotic Cement Combination.

Postoperative leukocyte counts are summarized in Table 6, while their temporal evolution is illustrated in Figure 4.

Mean leukocyte counts decreased progressively throughout the first postoperative week in all treatment groups.

No statistically significant differences were observed among treatment groups on postoperative Day 1 (*p* = 0.254). The gentamicin-only group presented the highest mean leukocyte count (12.74 ± 1.82 × 10^3^/μL), whereas lower values were observed in all combination therapy groups, ranging from 10.60 ± 1.31 × 10^3^/μL in the gentamicin–meropenem group to 11.83 ± 1.98 × 10^3^/μL in the gentamicin–clindamycin group.

On postoperative Day 3, leukocyte counts differed significantly among treatment groups (*p* = 0.048). The lowest mean value was observed in the gentamicin–tobramycin group (8.17 ± 1.72 × 10^3^/μL), closely followed by the gentamicin–meropenem (8.19 ± 2.84 ×10^3^/μL) and gentamicin–clindamycin (8.20 ± 2.44 × 10^3^/μL) groups. The gentamicin-only group maintained the highest leukocyte count (11.67 ± 1.54 × 10^3^/μL).

By postoperative Day 7, leukocyte counts had decreased in all treatment groups, and no statistically significant differences remained between regimens (*p* = 0.567). Mean leukocyte values ranged from 7.36 ± 1.45 × 10^3^/μL in the gentamicin–vancomycin group to 8.61 ± 1.93 × 10^3^/μL in the gentamicin–tobramycin group.

Table 4 summarizes postoperative inflammatory marker values on Day 1 following primary total hip arthroplasty. Significant between-group differences were observed for CRP and ESR (both *p* < 0.001), whereas leukocyte counts did not differ significantly (*p* = 0.254). Among the evaluated regimens, the gentamicin–meropenem group exhibited the lowest mean CRP values, while the gentamicin-only group demonstrated the highest CRP concentrations. The significant between-group differences in CRP and ESR were accompanied by large overall effect sizes (η^2^ = 0.896 and η^2^ = 0.918, respectively).

Table 5 presents inflammatory marker values on postoperative Day 3. CRP remained significantly different among treatment groups (*p* < 0.001), whereas ESR showed no significant between-group difference (*p* = 0.099). Leukocyte counts differed significantly (*p* = 0.048), with lower values generally observed among patients receiving dual-antibiotic bone cement formulations compared with gentamicin-loaded cement alone. The between-group difference in CRP was associated with a large effect size (η^2^ = 0.962), whereas the effects for ESR and leukocyte count were smaller (η^2^ = 0.223 and 0.266, respectively).

Table 6 summarizes inflammatory marker values on postoperative Day 7. Significant between-group differences persisted for CRP and ESR (both *p* < 0.001), whereas leukocyte counts no longer differed significantly (*p* = 0.567). The gentamicin–meropenem regimen remained associated with the lowest mean CRP and ESR values at the final postoperative assessment. Large between-group effect sizes were observed for CRP (η^2^ = 0.813) and ESR (η^2^ = 0.798), whereas the effect for leukocyte count was small (η^2^ = 0.090). Between-group comparisons were performed separately at each predefined postoperative time point, as the primary analytical objective was to compare treatment groups at each assessment rather than to formally evaluate within-patient longitudinal trajectories or group × time interactions.

Table 7 summarizes the statistical significance of between-group differences in postoperative inflammatory markers across the three evaluation time points. CRP demonstrated significant differences among the five antibiotic-loaded bone cement groups at all postoperative assessments (Days 1, 3, and 7; all *p* < 0.001), indicating a consistent effect of the antibiotic regimen on postoperative inflammatory responses. ESR showed significant differences on postoperative Days 1 and 7 (both *p* < 0.001), whereas no significant between-group difference was observed on Day 3 (*p* = 0.099). Leukocyte count demonstrated a different pattern, with a significant difference detected only on postoperative Day 3 (*p* = 0.048), while no significant differences were observed on Days 1 (*p* = 0.254) or 7 (*p* = 0.567). Overall, these findings suggest that CRP was the most sensitive marker for detecting differences between antibiotic-loaded bone cement formulations during the early postoperative period, whereas ESR and leukocyte count showed more limited discriminatory ability. Bonferroni-adjusted post-hoc analyses were performed to account for multiple pairwise comparisons between treatment groups. For CRP, the gentamicin-only group demonstrated significantly higher values than the dual-antibiotic groups at the evaluated postoperative time points after Bonferroni adjustment. Additional significant differences were observed between selected dual-antibiotic groups, particularly between the gentamicin–clindamycin and gentamicin–meropenem groups. For ESR, several pairwise differences remained statistically significant after Bonferroni adjustment on postoperative Days 1 and 7, whereas no significant pairwise differences were observed on Day 3. For leukocyte counts, no pairwise between-group comparison remained statistically significant after Bonferroni correction. These post-hoc findings were interpreted as secondary to the overall longitudinal repeated-measures analysis.

## 4. Discussion

The present prospective observational study evaluated the early postoperative inflammatory response following primary total hip arthroplasty using five different antibiotic-loaded bone cement (ALBC) formulations in patients with significant comorbidities and associated with an increased risk of periprosthetic joint infection (PJI). Although all treatment groups demonstrated the expected postoperative decline in inflammatory markers, significant differences were observed among antibiotic regimens. CRP consistently demonstrated significant between-group differences throughout the postoperative period, whereas ESR showed significant differences on postoperative Days 1 and 7, and leukocyte counts differed significantly only on Day 3. Among the evaluated regimens, the gentamicin–meropenem combination was associated with the lowest postoperative CRP and ESR values, suggesting a more favorable early inflammatory profile compared with the other antibiotic combinations.

Antibiotic-loaded bone cement has become an important adjunct in the prevention of PJI by providing high local antibiotic concentrations while minimizing systemic exposure. PMMA-based cement enables sustained local antibiotic release, particularly during the first postoperative days, when bacterial colonization of the implant is most likely to occur. Gentamicin remains the most frequently incorporated antibiotic because of its thermal stability, broad antimicrobial spectrum, and well-established elution characteristics. Nevertheless, combining gentamicin with additional antimicrobial agents has been proposed to broaden bacterial coverage and improve local antibiotic release through increased cement porosity and enhanced diffusion of antimicrobial agents [19,20,21].

In the present study, patients receiving gentamicin alone exhibited the highest CRP concentrations throughout the observation period and demonstrated slower normalization of postoperative inflammatory markers than patients receiving combination antibiotic regimens. Although gentamicin remains an effective prophylactic agent, these findings suggest that the addition of a second antibiotic may improve early control of postoperative inflammation. Similar observations have been reported in previous experimental and clinical studies demonstrating improved local antimicrobial activity and enhanced antibiotic elution with dual-antibiotic formulations [21,22,23,24].

Among the evaluated regimens, the gentamicin–meropenem group demonstrated the lowest CRP and ESR values by postoperative Day 7. Meropenem provides broad-spectrum activity against multidrug-resistant Gram-negative organisms and has attracted increasing interest for local application in complex orthopaedic infections [25]. Although its incorporation into PMMA remains technically demanding because of its relative thermal instability, previous in vitro studies have demonstrated satisfactory elution characteristics and preservation of antibacterial activity following incorporation into bone cement [26,27]. While the present study was not designed to evaluate infection prevention directly, the lower inflammatory marker values observed in this group represent an association and cannot establish superior antimicrobial or infection-preventive efficacy.

The gentamicin–clindamycin group demonstrated relatively higher inflammatory marker values during the early postoperative period compared with the other combination regimens. Clindamycin remains an important alternative for patients with β-lactam allergy and provides reliable activity against Gram-positive organisms, particularly staphylococci [23,28]. However, its clinical performance may vary according to the local microbial spectrum and resistance patterns, which could partially explain the less pronounced reduction in inflammatory markers observed in the present cohort [24,29]. The gentamicin–vancomycin and gentamicin–tobramycin combinations produced intermediate inflammatory profiles. Vancomycin continues to play a central role in patients at high risk for methicillin-resistant *Staphylococcus aureus* infection, but in the same time concerns persist regarding its limited elution from PMMA and potential effects on cement mechanical integrity [30]. The gentamicin–vancomycin combination did not demonstrate the lowest inflammatory marker values despite its broad antimicrobial coverage. One possible explanation relates to differences in antibiotic elution from PMMA. Vancomycin has a considerably larger molecular size than gentamicin, and physicochemical characteristics such as molecular weight, charge, solubility, and hydrophilicity can influence diffusion and release from the cement matrix. These factors may therefore have contributed to the observed differences between antibiotic combinations, although antibiotic elution was not directly assessed in the present study.

Tobramycin is frequently incorporated into antibiotic-loaded bone cement because of its established compatibility with PMMA and favorable local elution characteristics [20,21,31]. However, because gentamicin and tobramycin belong to the same antibiotic class and have substantially overlapping antibacterial spectra, the gentamicin–tobramycin regimen evaluated in the present study should not be interpreted as providing the same degree of complementary antimicrobial coverage as combinations involving antibiotics from different classes. Gentamicin is widely used in commercially available antibiotic-loaded bone cements, either alone or in combination with other antibiotics. Although gentamicin and tobramycin have similar mechanisms of action and substantially overlapping antibacterial spectra, their clinical use varies geographically, with gentamicin being more commonly used in European practice and tobramycin more frequently used in United States [32]. Although neither combination achieved inflammatory marker reductions comparable to those observed with gentamicin–meropenem, both demonstrated more rapid normalization than gentamicin alone, supporting the potential benefits of dual-antibiotic formulations in selected high-risk patients. An interesting finding was the progressive increase in ESR observed in the gentamicin-only group during the first postoperative week, despite the simultaneous decline in CRP. This apparent discrepancy may be explained by the different kinetics of these inflammatory biomarkers. ESR generally responds more slowly than CRP to an acute inflammatory stimulus and may remain elevated or continue to rise during the early postoperative period because it is influenced by changes in acute-phase plasma proteins and postoperative hematologic factors. Therefore, the ESR increase observed in the gentamicin-only group should not necessarily be interpreted as evidence of worsening inflammation. The mechanism underlying this finding cannot be established from the present data, and ESR should therefore be interpreted together with CRP and leukocyte count rather than as an isolated marker. Importantly, CRP and ESR are nonspecific markers of inflammation, and lower postoperative values should not be interpreted as direct evidence of superior infection prevention. In this study, these biomarkers reflect differences in the early postoperative inflammatory response rather than the clinical efficacy of a cement formulation in preventing PJI. The differences in inflammatory marker profiles observed among the antibiotic regimens are consistent with previous reports showing that the incorporation of multiple antibiotics into PMMA may influence antibiotic release and antimicrobial coverage. Enhanced porosity of the cement matrix facilitates antibiotic diffusion during the critical early postoperative period, potentially contributing to more effective bacterial suppression around the implant [22,23,24,33]. Nevertheless, the choice of antibiotics should always balance antimicrobial efficacy with preservation of the mechanical properties of the cement and should be guided by local microbiological epidemiology and individual patient risk factors [19,21]. Concerns regarding the potential development of antibiotic resistance must also be considered. Although theoretically possible, current evidence does not demonstrate a clear association between ALBC use and increased resistance when appropriate antibiotic selection and dosing are employed [34].

The findings of this study should be interpreted in light of certain limitations. First, the small sample size (seven patients per group) limited statistical power and generalizability. Second, the single-center observational design and non-randomized treatment allocation introduced potential selection bias and residual confounding. Third, differences in PMMA cement formulation (Palacos^®^ R + G vs. Simplex P^®^ with tobramycin) and antibiotic incorporation technique may have affected antibiotic distribution, porosity, and elution, potentially contributing to between-group differences. Because antibiotic elution was not directly measured and the sample size precluded stratification by cement type or preparation technique, differences in postoperative inflammatory biomarkers cannot be attributed solely to the antibiotic combinations. The seven-day follow-up prevented assessment of long-term outcomes, including periprosthetic joint infection, implant survival, and functional recovery. Finally, inflammatory biomarkers are surrogate measures and cannot establish the clinical superiority of one antibiotic regimen. Future studies should include larger randomized cohorts, standardized cement formulations and admixing protocols, direct assessment of antibiotic elution, and longer follow-up. The antibiotic doses were not equivalent across the investigated formulations. In particular, clindamycin was incorporated at 900 mg per 40 g PMMA, which is slightly lower than the 1 g per 40 g PMMA. Differences in antibiotic loading may influence local antibiotic elution and therefore represent an additional potential confounding factor when comparing postoperative inflammatory profiles among the investigated formulations. A higher clindamycin load might have resulted in a different inflammatory profile; however, this cannot be determined from the present data. The analysis was performed separately at each postoperative time point and did not formally account for within-patient correlations across repeated measurements; therefore, longitudinal group × time effects could not be assessed. Potential confounding by demographic, clinical, and perioperative factors cannot be excluded. The small sample size and limited availability of patient-level covariate data precluded reliable multivariable adjustment.

Despite these limitations, the present findings provide clinically relevant information regarding the early postoperative inflammatory response associated with different antibiotic-loaded bone cement formulations in high-risk patients undergoing primary total hip arthroplasty. The consistent reduction in CRP observed with combination therapies, particularly the gentamicin–meropenem regimen, supports the concept of individualized local antibiotic strategies tailored to patient characteristics and microbiological risk. Larger multicenter randomized studies with longer follow-up are warranted to determine whether these early biochemical differences translate into lower rates of periprosthetic joint infection and improved long-term clinical outcomes.

## 5. Conclusions

In this prospective observational study, different antibiotic-loaded PMMA formulations were associated with distinct early postoperative inflammatory biomarker profiles following primary THA. Gentamicin–meropenem was associated with comparatively lower CRP and ESR values at several assessed time points; however, these findings should not be interpreted as evidence of superior clinical efficacy or infection prevention. Given the small sample size and observational design, larger prospective studies with clinically confirmed PJI as an endpoint are needed to validate these findings.

## Figures and Tables

**Table 1:** Bone cement formulations and antibiotic content according to study group.

Group	Antibiotic Regimen	Bone Cement	Premixed Antibiotic Content	Manually Added Antibiotic
A	Gentamicin	Palacos^®^ R + G	Gentamicin 0.5 g/40 g PMMA	—
B	Gentamicin + Vancomycin	Palacos^®^ R + G	Gentamicin 0.5 g/40 g PMMA	Vancomycin 2 g/40 g PMMA
C	Gentamicin + Clindamycin	Palacos^®^ R + G	Gentamicin 0.5 g/40 g PMMA	Clindamycin 0.9 g/40 g PMMA
D	Gentamicin + Meropenem	Palacos^®^ R + G	Gentamicin 0.5 g/40 g PMMA	Meropenem 1 g/40 g PMMA
E	Gentamicin + Tobramycin	Simplex P^®^ with tobramycin	Tobramycin 1 g/40 g PMMA	Gentamicin 0.5 g/40 g PMMA

**Table 2:** Baseline demographic and clinical characteristics of patients according to antibiotic-loaded bone cement group.

Characteristic	Gentamicin (*n* = 7)	Genta + Clindamycin (*n* = 7)	Genta + Meropenem (*n* = 7)	Genta + Tobramycin (*n* = 7)	Genta + Vancomycin (*n* = 7)	*p*-Value
Age, years	59.9 ± 7.6	62.1 ± 5.9	62.9 ± 6.9	58.7 ± 5.7	57.6 ± 6.9	0.535
Female, *n* (%)	4 (57.1)	3 (42.9)	5 (71.4)	3 (42.9)	4 (57.1)	0.807
Male, *n* (%)	3 (42.9)	4 (57.1)	2 (28.6)	4 (57.1)	3 (42.9)	—
BMI, kg/m^2^	24.3 ± 2.1	24.5 ± 2.5	25.5 ± 2.7	24.8 ± 4.3	25.5 ± 2.7	0.908
ASA II, *n* (%)	7 (100)	7 (100)	7 (100)	7 (100)	7 (100)	—
Duration of intervention, min	58.6 ± 8.0	54.3 ± 7.3	62.1 ± 8.6	60.0 ± 8.7	54.3 ± 4.5	0.229
Relevant comorbidities, *n* (%)						
Type 2 diabetes mellitus	2 (28.6)	2 (28.6)	1 (14.3)	2 (28.6)	2 (28.6)	
Chronic kidney disease	2 (28.6)	1 (14.3)	1 (14.3)	1 (14.3)	1 (14.3)	
Hypertension	2 (28.6)	3 (42.9)	4 (57.1)	3 (42.9)	4 (57.1)	
Iron-deficiency anemia	1 (14.3)	1 (14.3)	1 (14.3)	1 (14.3)	0 (0)	

Values are presented as mean ± standard deviation (SD) for continuous variables and *n* (%) for categorical variables. Continuous variables were compared using one-way ANOVA. ASA, American Society of Anesthesiologists; BMI, body mass index.

**Table 3:** Repeated-Measures Analysis of Postoperative Inflammatory Biomarkers According to Treatment Group and Time.

Outcome	Group Effect	Time Effect	Group × Time
**CRP**	*p* < 0.001	*p* < 0.001	*p* < 0.001
**ESR**	*p* < 0.001	*p* < 0.001	*p* < 0.001
**Leukocytes**	*p* = 0.071	*p* < 0.001	*p* < 0.001

Note: *p*-values represent the overall effects of treatment group, postoperative time (Days 1, 3, and 7), and the group × time interaction in the longitudinal repeated-measures model. A two-sided *p* < 0.05 was considered statistically significant.

**Table 4:** Postoperative inflammatory markers on Day 1 according to antibiotic-loaded bone cement group, with one-way ANOVA results, 95% confidence intervals, and effect sizes (η^2^).

Group	CRP, Mean ± SD (95% CI)	ESR, Mean ± SD (95% CI)	Leukocytes, Mean ± SD (95% CI)
Gentamicin	127.81 ± 11.70 (116.99–138.63)	54.33 ± 4.42 (50.24–58.42)	12.74 ± 1.82 (11.06–14.42)
Gentamicin + Clindamycin	102.87 ± 2.36 (100.69–105.05)	77.74 ± 1.74 (76.13–79.35)	11.83 ± 1.98 (10.00–13.66)
Gentamicin + Meropenem	83.50 ± 2.32 (81.35–85.65)	68.67 ± 1.98 (66.84–70.50)	10.60 ± 1.31 (9.39–11.81)
Gentamicin + Tobramycin	90.91 ± 1.98 (89.08–92.74)	73.01 ± 1.87 (71.28–74.74)	10.64 ± 2.99 (7.87–13.41)
Gentamicin + Vancomycin	97.17 ± 1.84 (95.47–98.87)	73.97 ± 2.15 (71.98–75.96)	10.69 ± 2.18 (8.67–12.71)

*p*-values obtained using one-way ANOVA. *p* < 0.05 considered statistically significant. ANOVA: CRP *p* < 0.001, η^2^ = 0.896; ESR *p* < 0.001, η^2^ = 0.918; leukocytes *p* = 0.254, η^2^ = 0.158. Note: Values are presented as mean ± standard deviation (95% confidence interval). Between-group comparisons were performed using one-way ANOVA. Eta-squared (η^2^) represents the overall between-group effect size. CRP, C-reactive protein; ESR, erythrocyte sedimentation rate.

**Table 5:** Postoperative inflammatory markers on Day 3 according to antibiotic-loaded bone cement group, with one-way ANOVA results, 95% confidence intervals, and effect sizes (η^2^).

Group	CRP, Mean ± SD (95% CI)	ESR, Mean ± SD (95% CI)	Leukocytes, Mean ± SD (95% CI)
Gentamicin	86.39 ± 4.35 (82.37–90.41)	63.61 ± 11.40 (53.07–74.15)	11.67 ± 1.54 (10.25–13.09)
Gentamicin + Clindamycin	64.27 ± 1.37 (63.00–65.54)	67.06 ± 1.77 (65.42–68.70)	8.20 ± 2.44 (5.94–10.46)
Gentamicin + Meropenem	52.76 ± 1.68 (51.21–54.31)	60.89 ± 1.52 (59.48–62.30)	8.19 ± 2.84 (5.56–10.82)
Gentamicin + Tobramycin	58.76 ± 1.39 (57.47–60.05)	60.79 ± 1.66 (59.25–62.33)	8.17 ± 1.72 (6.58–9.76)
Gentamicin + Vancomycin	58.86 ± 2.42 (56.62–61.10)	66.46 ± 2.30 (64.33–68.59)	9.00 ± 3.15 (6.09–11.91)

*p*-values obtained using one-way ANOVA. *p* < 0.05 considered statistically significant. ANOVA: CRP *p* < 0.001, η^2^ = 0.962; ESR *p* = 0.099, η^2^ = 0.223; leukocytes *p* = 0.048, η^2^ = 0.266. Note: Values are presented as mean ± standard deviation (95% confidence interval). Between-group comparisons were performed using one-way ANOVA. Eta-squared (η^2^) represents the overall between-group effect size. CRP, C-reactive protein; ESR, erythrocyte sedimentation rate.

**Table 6:** Postoperative inflammatory markers on Day 7 according to antibiotic-loaded bone cement group, with one-way ANOVA results, 95% confidence intervals, and effect sizes (η^2^).

Group	CRP, Mean ± SD (95% CI)	ESR, Mean ± SD (95% CI)	Leukocytes, Mean ± SD (95% CI)
Gentamicin	59.17 ± 13.02 (47.13–71.21)	66.26 ± 8.56 (58.34–74.18)	8.46 ± 1.13 (7.41–9.51)
Gentamicin + Clindamycin	35.00 ± 2.42 (32.76–37.24)	50.39 ± 1.20 (49.28–51.50)	7.79 ± 0.98 (6.88–8.70)
Gentamicin + Meropenem	25.93 ± 1.89 (24.18–27.68)	43.50 ± 3.03 (40.70–46.30)	8.49 ± 2.40 (6.27–10.71)
Gentamicin + Tobramycin	33.59 ± 1.15 (32.53–34.65)	45.66 ± 2.55 (43.30–48.02)	8.61 ± 1.93 (6.83–10.39)
Gentamicin + Vancomycin	28.77 ± 2.66 (26.31–31.23)	47.43 ± 2.66 (44.97–49.89)	7.36 ± 1.45 (6.02–8.70)

*p*-values obtained using one-way ANOVA. *p* < 0.05 considered statistically significant. ANOVA: CRP *p* < 0.001, η^2^ = 0.813; ESR *p* < 0.001, η^2^ = 0.798; leukocytes *p* = 0.567, η^2^ = 0.090. Note: Values are presented as mean ± standard deviation (95% confidence interval). Between-group comparisons were performed using one-way ANOVA. Eta-squared (η^2^) represents the overall between-group effect size. CRP, C-reactive protein; ESR, erythrocyte sedimentation rate.

**Table 7:** Summary of one-way ANOVA *p*-values comparing inflammatory markers among antibiotic-loaded bone cement groups at each postoperative time point.

Inflammatory Marker	Day 1	Day 3	Day 7
CRP	**<0.001**	**<0.001**	**<0.001**
ESR	**<0.001**	0.099	**<0.001**
Leukocyte count	0.254	**0.048**	0.567

## Data Availability

The data that support the findings of this study are available from the corresponding author upon reasonable request.

## Abbreviations

PJI	Periprosthetic joint infection
ALBC	Antibiotic-loaded bone cement
CRP	C-reactive protein
ESR	Erythrocyte Sedimentation Rate
CBC	Complete Blood Count
THA	Total Hip Arthroplasty
MRSA	Methicillin-resistant *Staphylococcus aureus*
PMMA	Polymethyl Methacrylate
MMA	Monomer Liquid
ANOVA	Analysis of Variance
